# Medical isotope production, research reactors and their contribution to the global xenon background

**DOI:** 10.1007/s10967-018-6128-2

**Published:** 2018-08-25

**Authors:** Ian Hoffman, Rodney Berg

**Affiliations:** Health Canada, Radiation Protection Bureau, 775 Brookfield Rd, Ottawa, K1A 1C1 Canada

**Keywords:** Medical isotope production, Reactor effluent, Radioxenon, CTBT

## Abstract

The Comprehensive Nuclear-Test-Ban Treaty (CTBT) bans the testing of nuclear explosive devices underground, in the atmosphere and underwater. Two main technologies, radionuclide and seismo-acoustic monitoring, are deployed in the International Monitoring System used for the verification of the CTBT. Medical isotope production from fission-based processes is the dominant contributor to a worldwide background of radioxenon. This background can make the discrimination of nuclear tests from legitimate nuclear activities very challenging. Even if emissions from medical isotope producers experienced a large reduction, there remain other important sources of radioxenon that contribute to the global background such as research reactors and nuclear power plants. Until recently, the largest producer of medical isotopes was located in Canada, at the Canadian Nuclear Laboratories (CNL) facility. The characterization of CNL emissions and its research reactor can provide valuable information for effective verification of the CTBT.

## Introduction

The Comprehensive Nuclear-Test-Ban Treaty (CTBT) bans the testing of nuclear explosive devices underground, in the atmosphere, and underwater. The Comprehensive Nuclear-Test-Ban Treaty Organization (CTBTO) is the international organization responsible for operating the global International Monitoring System (IMS) and provides an initial analysis of all IMS data through the International Data Centre (IDC). For a well-contained underground nuclear test, very little if any, particulate matter escapes to the atmosphere, with the result that the only radionuclide signature may be radioactive noble gases [1, 2]. For CTBT verification purposes, the noble gases monitored include the xenon isotopes ^133^Xe (*t*_1/2_ = 5.243 d) and ^135^Xe (*t*_1/2_ = 9.10 h) and the nuclear isomers ^131m^Xe (*t*_1/2_ = 11.9 d) and ^133m^Xe (*t*_1/2_ = 2.19 d). However, medical isotope production has a large influence on the CTBT radioxenon monitoring network [3–5]. Chemical and physical processes involved in medical isotopes production creates large quantities of radioxenon leading to difficulty in event discrimination using the Multi-Isotope Ratio Chart (MIRC) method [1]. The production process for medical isotopes generally uses short irradiation times (typically several days) of nuclear targets that when dissolved release radioxenon in similar isotopic ratios to a nuclear explosion if they are promptly vented to the atmosphere.

The aim of this study is to examine the impact on (background) radioxenon observations in the CTBTO IMS due to medical isotope production at the Canadian Nuclear Laboratories (CNL) facility in Chalk River, Ontario. This includes a characterization of the IMS impact due to changes in the operational state of the irradiation reactor. This reactor has ceased medical isotope production in October 2016, but remains an active research reactor.

Medical isotope production, in particular the production of ^99m^Tc which is used as a medical imaging tracer, has predominantly relied on the fission of highly enriched uranium targets [6]. The short irradiation times, typically 5–7 days in a high-flux reactor [6], are very different from the typical xenon signatures released from commercial power reactors in which the fuel remains in the reactor core for more than a year.

Past studies of medical isotope production have typically considered releases solely from processes related to the manufacture of radioisotopes [4, 5, 7–9]. These include the chemical processes (dissolution in an acid or alkaline solution) performed on the target material as well as releases from physical processes, such as the cementation of the radioactive waste materials. Although medical isotope production is the dominant contributor to worldwide radioxenon atmospheric inventories, this study will examine and provide the first characterization of radioxenon emissions due to the research reactor used in the production of medical isotopes.

## Material

**Fig. 1:**
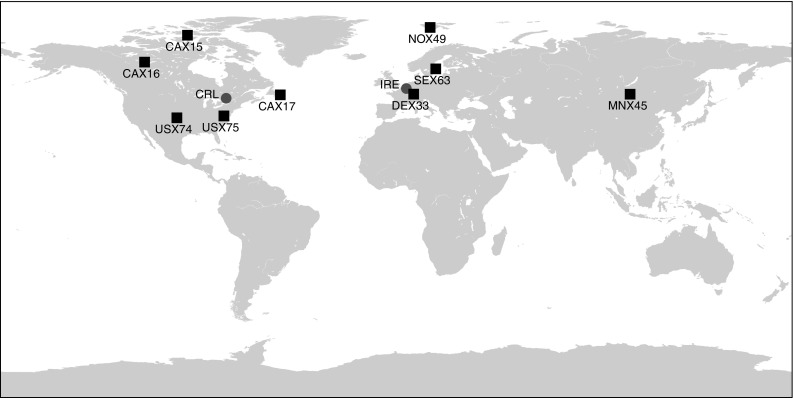
Locations of Radioxenon Monitoring Sites of the CTBTO IMS used for this study. The two medical isotope producers are marked with a circle, while the noble gas monitoring locations are marked with a square. MNX45 data was only integrated into the total assessment (Fig. 7)

The radioxenon background was examined by using emissions data provided by medical isotope facility operators combined with observations of radioxenon from the IMS of the CTBTO. Two of the biggest medical isotope production facilities worldwide, Institute for Radioelements (IRE) and CNL, graciously supplied data that was used to assess the impact of medical isotope production, while CNL data was used to assess the impact of a high-flux research reactor, the National Research Universal (NRU), on the global radioxenon background. For the noble gas monitoring sites, the following sites were specifically considered: CAX14, CAX15, CAX17, DEX33, SEX63, NOX49, USX74, USX75, as well as the total aggregated data (which included MNX45). The specific monitoring locations are shown in Fig. 1 and tabulated in Table 1.

**Table 1:** IMS monitoring locations used in this study

IMS station code	Location
CAX15	Resolute Bay, NU, Canada
CAX16	Yellowknife, NT, Canada
CAX17	St. John’s, NL, Canada
DEX33	Schauinsland, Germany
MNX45	Ulaanbataar, Mongolia
NOX49	Spitsbergen, Norway
SEX63	Stockholm, Sweden
USX74	Ashland, KA, USA
USX75	Charlottesville, VA, USA

The availability of data restricted this study to the Northern Hemisphere, but the outcomes are equally applicable in the Southern Hemisphere. The specific monitoring periods for reactor/medical isotope production considered for each observation site are given in Table 2. The *Normal Operational* period included all available observations beginning in 2003-07-22 to the end of medical isotope production in 2016-10-31, but with any observations from the period 2011-03-11 through 2011-08-31 excluded due to interferences from the Fukushima reactor accident. The gradual commissioning of the IMS network means that some sites have a much longer observation history than others.

**Table 2:** Relevant time periods for global radioxenon atmospheric inventory

Period	Interval start	Interval end
Complete reactor shutdown	2007-11-23	2007-12-16
	2009-05-31	2010-08-18
Medical isotope production suspended	2016-10-31	2017-02-18
Fukushima excluded data	2011-03-11	2011-08-31

All other times from 2003-07-22 onwards were considered normal reactor operations

### Stack monitoring

The stack monitoring system at CNL has an Alpha Spectra Inc. 614/2 photomultiplier connected to a NaI(Tl) crystal that is 1.5 inches in diameter and 1 inch long and an Ortec 266 14-pin base. Pulses were amplified by a Ortec 863 four channel amplifier which was connected to a Ortec 425A delay module followed by an Ortec 583B CFD/SCA and Ortec 449 log/lin ratemeter. Reporting was handled by a Eurotherm 6100 and was configured to report in four regions as shown in Table 3. The system was designed to record count rates from 10 to 5E5 cps. Counts recorded by the system were integrated into 30 min intervals.

**Table 3:** Four channel amplifier configuration of the stack monitoring system at CNL

Channel	Energy range (keV)
1	50–120
2	120–300
3	300–662
4	> 662

In addition to the standard stack monitoring equipment, principally used for daily release limited monitoring, CNL has also installed a High-Purity Germanium (HPGe) monitoring equipment to better characterize their emissions. A Canberra Falcon 5000^®^ was used as it was convenient due to it being portable and electrically cooled. A 1-h spectral acquisition period was used and the resulting spectra were analysed using Unisampo/Shaman to evaluate the xenon emissions from the NRU research reactor.

The emissions data from the main reactor stack and the molybdenum production stack have been collected by AECL with further processing performed by Health Canada before storing in a database. This database of emission data will soon be available upon request to the CTBTO.

## Method

This paper examines the broad scale impact of medical isotope production and research reactors on radioxenon background levels. To understand the distribution of atmospheric Northern Hemisphere radioxenon, three specific operational domains of the research reactor at CNL located in Chalk River, Ontario were considered. The first domain, *Normal Operations*, is during periods of normal operations. In this domain, the research reactor is operating and medical isotope production is active. In this mode, CNL would dissolve fuel rods typically two or three times weekly. Acid dissolution of these rods to recover ^99^Mo is what produces the majority of radioxenon emission from the site, when the effluent gases are released via a dedicated stack to the atmosphere. This domain supplies the majority of the data used in the study. The second domain, *Complete Shutdown*, is the case where the reactor has ceased functioning for a significant duration of time (more than a week). Consequently, medical isotope production is also suspended. These outages were an indication of significant maintenance activities being carried out. The final case, *Medical Isotope Shutdown*, is the current operational mode where the research reactor is operational but without the production of medical isotopes. In all cases, a suitable buffer period of 2 weeks was used to allow the atmospheric inventory to build up or dissipate as appropriate.

The IMS observing stations can also be classified into two different groups. The majority of stations are located in temperate areas at latitudes similar to the emission site. However, there are two arctic observing sites which may show different behaviour as they are located in the Polar atmospheric cell rather than in the mid-latitude cell.

Atmospheric cells such as the mid-latitude and polar cell are generally separated from each other in terms of their respective bulk air masses. This separation means that observations in these polar locations may have different behaviour as the atmospheric transport processes are more complex as the radioxenon plume must traverse cell boundaries.

### Xenon ratios

Ratios of xenon species are important indicators of nuclear tests [1, 2]. With the cessation of medical isotope production, an examination of the ratios of xenon emissions from research reactors is an important consideration for CTBT screening.

### Sampling time

The Systéme de Prélèvements et d’Analyse en Ligne d’Air pour quantifier le Xénon (SPALAX) located in Ottawa has undergone changes in its sampling period to optimize its performance. The collection time has varied from the standard 24 h period to using 12 h and 8 h. Improvements in temporal sampling resolution greatly assist with geolocation of sources and can provide better estimates of release quantities as the sample is less diluted from air unrelated to the emission site or time period of interest. Complete details on the SPALAX system and its operations can be found in [3].

## Results and discussion

**Fig. 2:**
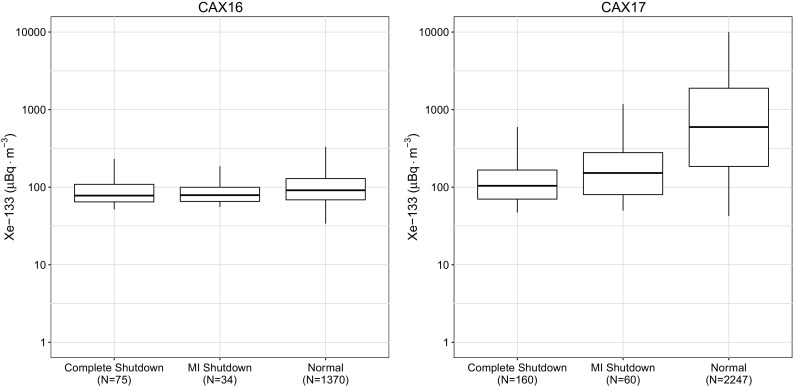
Observations at the North American CTBT sites located in Yellowknife, NT (CAX16) and St. John’s, NL (CAX17). The number of samples, *N*, for each box plot is shown in the label

**Fig. 3:**
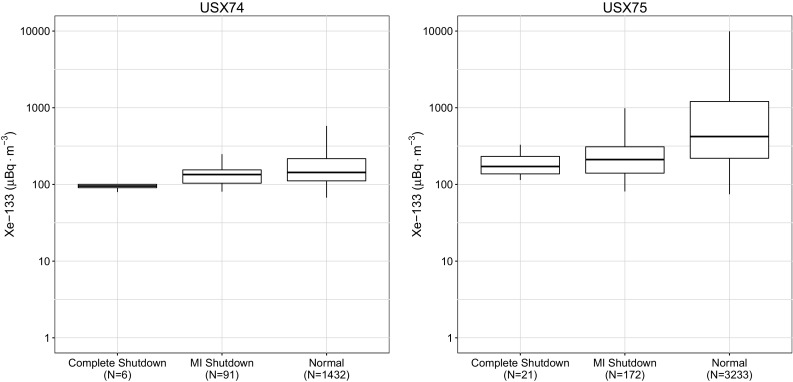
Observations at the North American CTBT sites located in Ashland, KS (USX74) and Charlottesville, VA (USX75). The number of samples, *N*, for each box plot is shown in the label

**Fig. 4:**
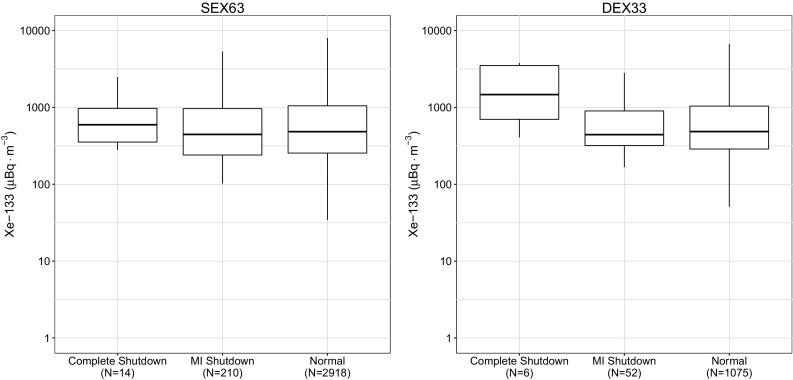
Observations at the European CTBT sites located in Stockholm, Sweden (SEX63) and Schauinsland, Germany (DEX33). The number of samples, *N*, for each box plot is shown in the label

For the selected IMS stations, a series of box plots were generated for each of the three reactor operational states as discussed in the “Material” section. The location of the monitoring sites fell into two different geographical domains. The majority of the sites were mid-latitude or located in the mid-latitude atmospheric cell, while there were two sites that were located at high latitudes, in the polar circulation cell. Both the CNL and IRE reactor facilities are located in the mid-latitude region. The emissions data was aggregated by station and summarized as shown in Figs. 2, 3, 4 and 6.

As expected the median xenon concentrations observed worldwide were typically greater during medical isotope production at CNL, and the overall distribution was broader for all the sites in North America. During the complete shutdown period, the observations in Europe showed an increase in ^133^Xe as shown in Fig. 4, which suggests that production at the IRE facility increased to make up for the lack of production at CNL.

**Fig. 5:**
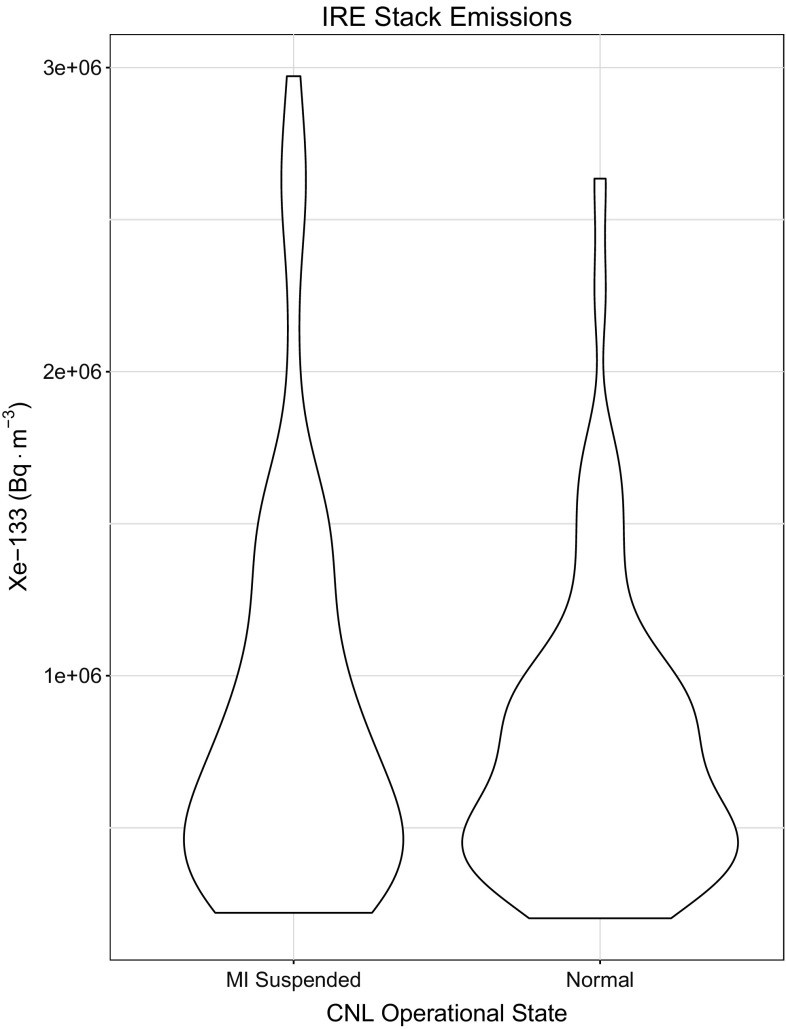
A violin plot showing the distribution and kernel density of 12 averaged IRE emissions when CNL was operating normally and when medical isotope production was suspended at the plant, but research was still being conducted. The kernel density, or y-axis profile, shows an estimate of the frequency of occurrence of emissions in the population. Although the profiles of both IRE states are similar, there appears to be some evidence that there is an increase in emissions when CNL was not producing medical isotopes

This finding is somewhat confirmed by examining 2 month-long periods of stack data from the IRE facility. Total emissions from the stack were approximately 10% greater during the month that CNL was not producing medical isotopes. Further examination of the behaviour of the IRE facility when CNL was not producing was performed through the generation of a violin plot as shown in Fig. 5. Typically, the emissions are very similar regardless of the operating state of CNL, however there is some evidence of increased emissions as the high-tail of the kernel density is both broader and longer indicating more frequent occurrences of increased emissions.

**Fig. 6:**
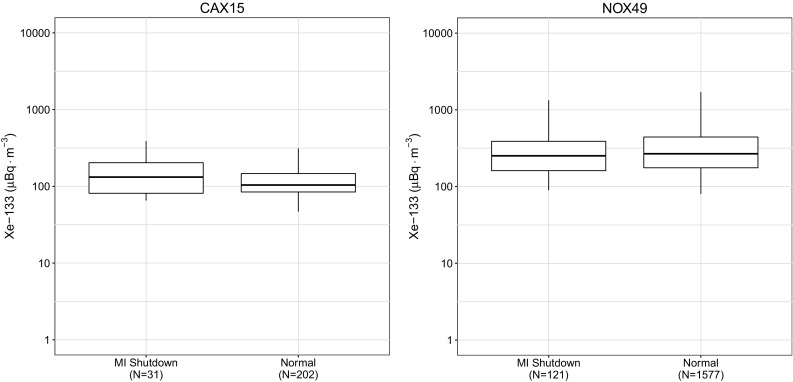
Observations at the noble gas site located in the high arctic, Resolute Bay (CAX15) and the CTBT site located in Spitsbergen, Norway (NOX49). The number of samples, *N*, for each box plot is shown in the label

Radioxenon monitoring sites in the arctic behave very differently from those at temperate latitudes, probably due to the separation of air masses in the mid-latitude and polar atmospheric zones. For the majority of monitoring sites (i.e. the mid-latitude sites) used in this study, zonal flow is sufficient to disperse the xenon plume from the emission location to the receptor site, but for the two arctic sites there must be sufficient meridional flow to carry the plume beyond the mid-latitude cell boundary northward into the arctic. This Fig. 6 shows the same aggregated observational data from the two monitoring sites located in the arctic. These two sites have somewhat inconsistent behaviour in their response to CNL operations. One possible explanation for the overall behaviour of the arctic stations is that as the arctic air is somewhat separate from the air at the emission sites, it may tend to act as a broad-scale (non-source specific) long-term collector of radioxenon, but further work using atmospheric dispersion models would be needed to confirm this hypothesis.

**Fig. 7:**
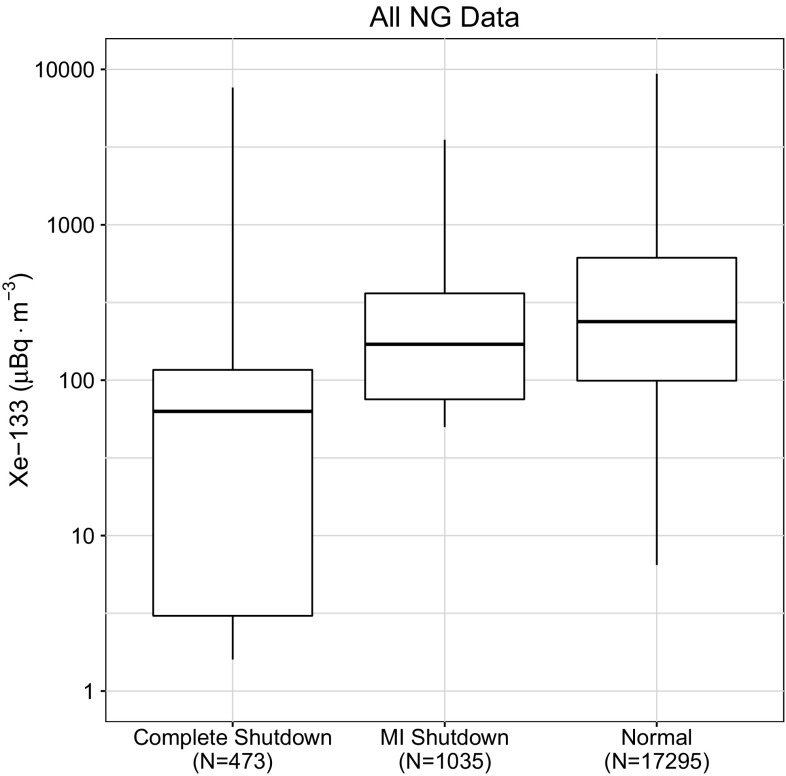
Complete data set aggregated and plotted according to the three domains as described in “Results and discussion” section. The number of samples, *N*, for each box plot is shown in the label

Aggregating all station observation data from this study gives the box plot shown in Fig. 7, which follows the trends discussed in the first North American plots, as there is a much longer observation history at the North American sites than those located in Europe and Asia. The global median xenon concentrations do not vary much when CNL is producing medical isotopes or only operating their research reactor. The limited reduction in atmospheric radioxenon is partially due to increased production elsewhere. Unless the entire facility becomes non-operational, the atmospheric levels of radioxenon are still significant from a total radioxenon inventory perspective.

**Fig. 8:**
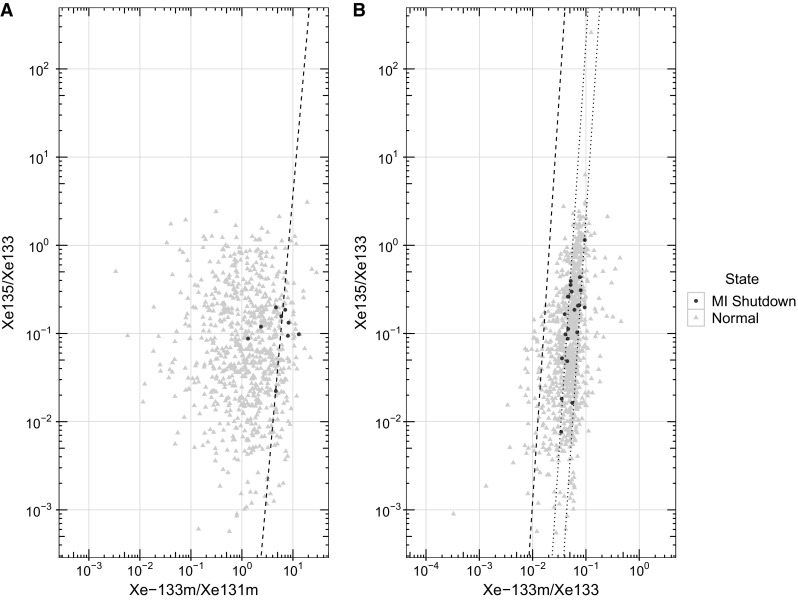
**a** Four isotope and **b** three isotope MIRC plots of data from the SPALAX located in Ottawa. The screening line (dashed) originally proposed in [1] was recreated. Typical nuclear power plant operations show up left of the dashed line while ratios on the right of the dashed line are more characteristic of a nuclear explosion. The dotted lines in the three ratio plot appear to show a behaviour that is most likely related to activities onsite, but further investigation is required

Finally, the ratios produced by research reactors were examined using data from the SPALAX in Ottawa. Analysis of noble gas data can be difficult as there is a covariance between the ^133m^Xe and the ^131m^Xe activity concentrations. This covariance also makes it more challenging to identify ^131m^Xe in particular as it has a low intensity gamma, 1.95%, at 233.221 keV. This low-yield gamma makes identification difficult so the x-ray at 29.781 keV can be used if the primary gamma line, 233.221 keV, of ^133m^Xe is identified. To increase the population of the limited data available, if the primary gamma line for ^133m^Xe was found, the weighted concentration of ^131m^Xe was used. This represents the residual signal left after the ^133m^Xe peak was fit. All other concentrations were based upon the primary line activity of the respective nuclide. The resulting three and four isotope MIRC plots are shown in Fig. 8.

Several important findings based upon these figures are worth discussing further. The first important finding is that the research reactors used for medical isotope production themselves are important contributors to the radioxenon background as can be seen by examining the differences in median and the degree of overlap of the Interquartile Range (IQR) of the North American IMS observations and the globally aggregated data. Since the cessation of medical isotope production there has also been an increase in the frequency and amount of ^135^Xe observed in Ottawa, ON. This enhancement increases the difficulty in discrimination as observations of this isotope indicate a relatively fresh fission process.

At many sites, the median activity concentration of ^133^Xe barely changes between normal operations and during periods when medical isotope production was suspended. The radioxenon background levels hemispherically respond predictably to the operational state of medical isotope production. If a producer stops producing in one region, a greater background was observed in the region of other producers.

The second important finding is that sites in the polar region do not necessarily behave as those in the mid-latitude. Although there are a limited number of samples, CAX15 had a much broader IQR and greater median value after medical isotope production ceased at CNL. Further study with more observations and the use of atmospheric dispersion models is required to understand this result.

Lastly, in terms of isotopic composition, research reactors pose a discrimination challenge as many of the remote observations fall in the nuclear explosion domain whether you look at three or four isotopes. Conducting screening using a purely isotopic ratio formulation would result in many false positive events. A more sophisticated approach, perhaps including information on facility activities or on-site stack emissions, would be required for CTBT purposes in the vicinity of high flux research reactors to reduce the false positive rate.

### Research reactor inventory

The International Atomic Energy Agency (IAEA) Research Reactor Database (RRDB) classifies research reactors into low (< 10^12^ n cm^−2^ s^−1^), medium (≥ 10^12^ n cm^−2^ s^−1^ and < 10^14^ n cm^−2^ s^−1^) and high (≥ 10^14^ n cm^−2^ s^−1^) flux categories. The overall distribution of operating and temporarily shut down reactors is given in Table 4. The BR2 reactor (4th largest flux worldwide) used to bombard targets for future medical isotope production at IRE, and the NRU reactor used by CNL (14th largest flux worldwide) belong in the high flux category.

**Table 4:** Classification of research reactors by thermal flux

Category	Number
Low	109
Medium	63
High	124

This table shows the number of reactors worldwide in each category that are currently operating or in temporary shut down mode

The large number of high flux reactors poses a potential problem for CTBT verification, depending on the nature of the activities at the research reactor. Short irradiation periods of nuclear materials pose the greatest problem for discrimination if the any resulting radioxenon is released to the atmosphere. Considering that there are currently 124 high flux reactors in operation or temporary shutdown worldwide with 6 more planned or under construction according to the RRDB, characterization of the facilities may be an important activity for effective CTBT verification. Furthermore, the worldwide reactor population includes 95 Isotope Production Facilities (IPF) of which 36 are high flux reactors. IPF emissions are well known to increase the difficulty of source discrimination and may require emissions abatement for effective screening [8].

## Conclusion

Although medical isotope production dominates the global radioxenon background, analysis of the emissions of the underlying research reactors show that they are also significant contributors to the radioxenon background and may become increasing important in CTBT monitoring if emissions from medical IPF decline. Observations at IMS sites show that the high flux reactor at CNL is typically responsible for roughly 1–5% of their total atmospheric emission inventory. This quantity of emissions would place them as one of the larger sources of radioxenon globally, so they remain a very important source of radioxenon to understand.

Research reactors also pose problems in terms of the composition of their emissions. Examination of emissions from the CNL facility when it was operated as a research reactor and no medical isotope production was occurring shows that in many cases their emissions are more characteristic of a nuclear explosion source than a civilian power reactor. Screening these emissions on a purely isotopic composition basis does not appear possible. This behaviour depends on the nature of the activities that are occurring at the facility. As these facilities often perform a variety of experiments, there will be some dependence on the type of work being conducted, which means understanding research reactor processes will be increasingly important in the context of CTBT characterization. The inconsistent operational schedules of research reactors could potentially make xenon ratio analysis difficult if not impossible for use as an event discrimination tool unless additional information from the site operator is available.
